# Supplementary material for: Spectroelectrochemical Properties and Catalytic Activity in Cyclohexane Oxidation of the Hybrid Zr/Hf-Phthalocyaninate-Capped Nickel(II) and Iron(II) tris-Pyridineoximates and Their Precursors
Source: Molecules. 2021 Jan 11;26(2):336. doi: 10.3390/molecules26020336 (PMC7827310; doi:10.3390/molecules26020336)
Supplement: Supplementary file 1 [file molecules-26-00336-s001.pdf]

Supplementary Material

for

Spectroelectrochemical Properties and Catalytic Activity in Cyclohexane Oxidation of the Hybrid Zr/Hf-Phthalocyaninate-Capped Nickel(II) and Iron(II) tris-Pyridineoximates and their Precursors

Yan Z. Voloshin [1,2], Semyon V. Dudkin [1], Svetlana A. Belova [1], Daniel Gherca [3], Dumitru Samohvalov [3], Corina-Mihaela Manta [3], Maria-Andreea Lungan [3], Samuel M. Meier-Menches [4], Peter Rapta [5,*], Denisa Darvasiová [5], Michal Malček [5], Armando J. L. Pombeiro [6], Luísa M. D. R. S. Martins [6,*] and Vladimir B. Arion [7,*]

[1] Nesmeyanov Institute of Organoelement Compounds of the Russian Academy of Sciences, 119991, Moscow, Russia; voloshin@ineos.ac.ru (Y.Z.V); sdudkin@ineos.ac.ru (S.V.D); savkinasveta91@mail.ru (S.A.B)
[2] Gubkin Russian State University of Oil and Gas (National Research University), 119991 Moscow, Russia
[3] Sara Pharm Solutions S.R.L., 266-268 Calea Rahovei, Bucharest-5, Romania; daniel_gherca@yahoo.com (D.G); dumitru.samohvalov@sara-pharm.com (D.S.); corina.manta@sara-pharm.com (C-M.M); maria-andreea.lungan@sara-pharm.com (M-A.L)
[4] Department of Analytical Chemistry, University of Vienna, Währinger Strasse 38, A-1090 Vienna, Austria; samuel.meier@univie.ac.at (S.M.M.-M)
[5] Institute of Physical Chemistry and Chemical Physics, Faculty of Chemical and Food Technology, Slovak University of Technology in Bratislava, Radlinského 9, SK-812 37 Bratislava, Slovakia; denisa.darvasiova@stuba.sk (D.D); michal.malcek@stuba.sk (M.M); peter.rapta@stuba.sk (P.R)
[6] Centro de Química Estrutural, Instituto Superior Técnico, Universidade de Lisboa, Av. Rovisco Pais, 1049-001 Lisboa, Portugal; pombeiro@tecnico.ulisboa.pt (A.J.L.P); luisammartins@tecnico.ulisboa.pt (L.M.D.R.S.M)
[7] Institute of Inorganic Chemistry, University of Vienna, Währinger Strasse 42, A-1090 Vienna, Austria; vladimir.arion@univie.ac.at (V.B.A)

Content

IR spectra of **9–14** (Figures S1–S6) S3

Additional ESI mass spectra with associated fragmentation experiments for **13** and **14**, **11** and **12** and **9** and **10** (Figures S7–S9) S9

CVs for **9–12** (Figure S10) S12

DFT spin density distributions in double reduced species of **9–12** (Figure S11) S13

UV-vis-NIR spectroelectrochemistry of 11 and 12 in the region of the first and the second reduction peaks (Figure S12) S14

Spin density distributions in mono-oxidized species of **9–12** (Figure S13) S15

CVs of precursors **13–16** (Figure S14) S16

EPR spectra and spin density distributions in mono-reduced and mono-oxidized precursor **15** (Figure S15) S17

Frontier orbitals of **13** (Figure S16) S18

Evolution of UV-vis-NIR spectra upon CV of **14** in the region of the first eduction peak (Figure S17) S19

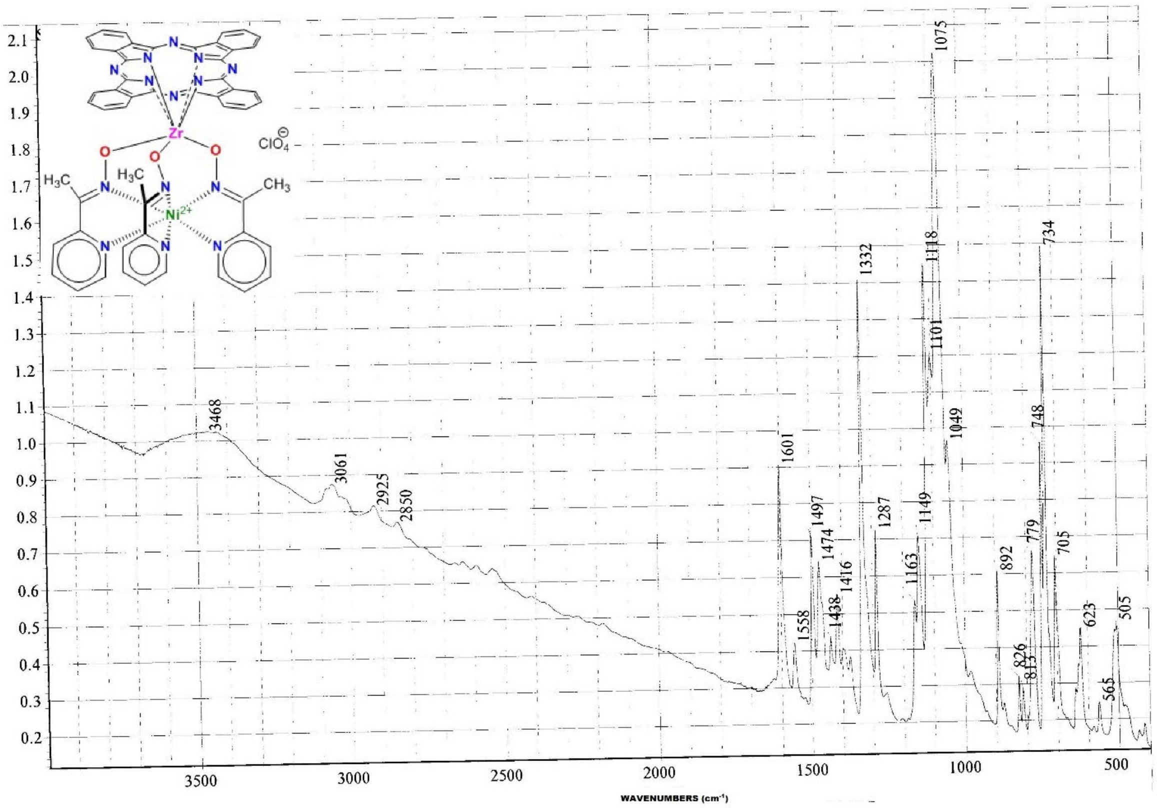

Figure S1. IR spectrum of **9**.

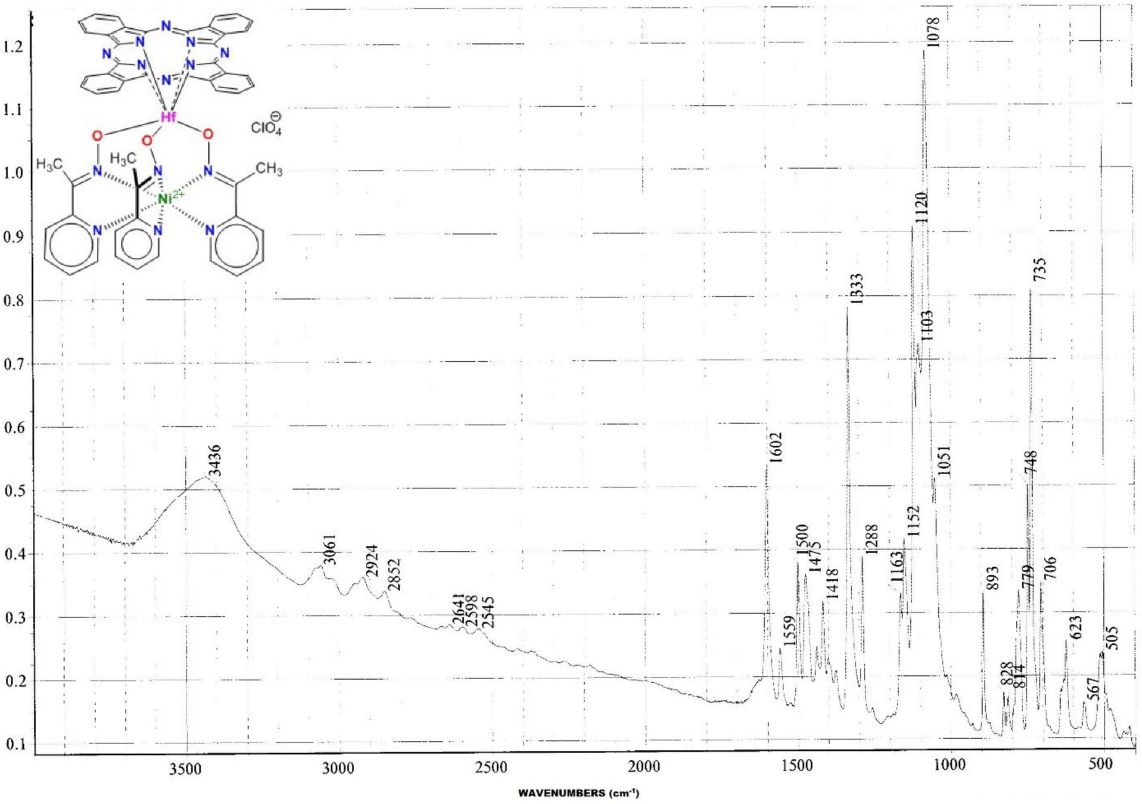

Figure S2. IR spectrum of **10**.

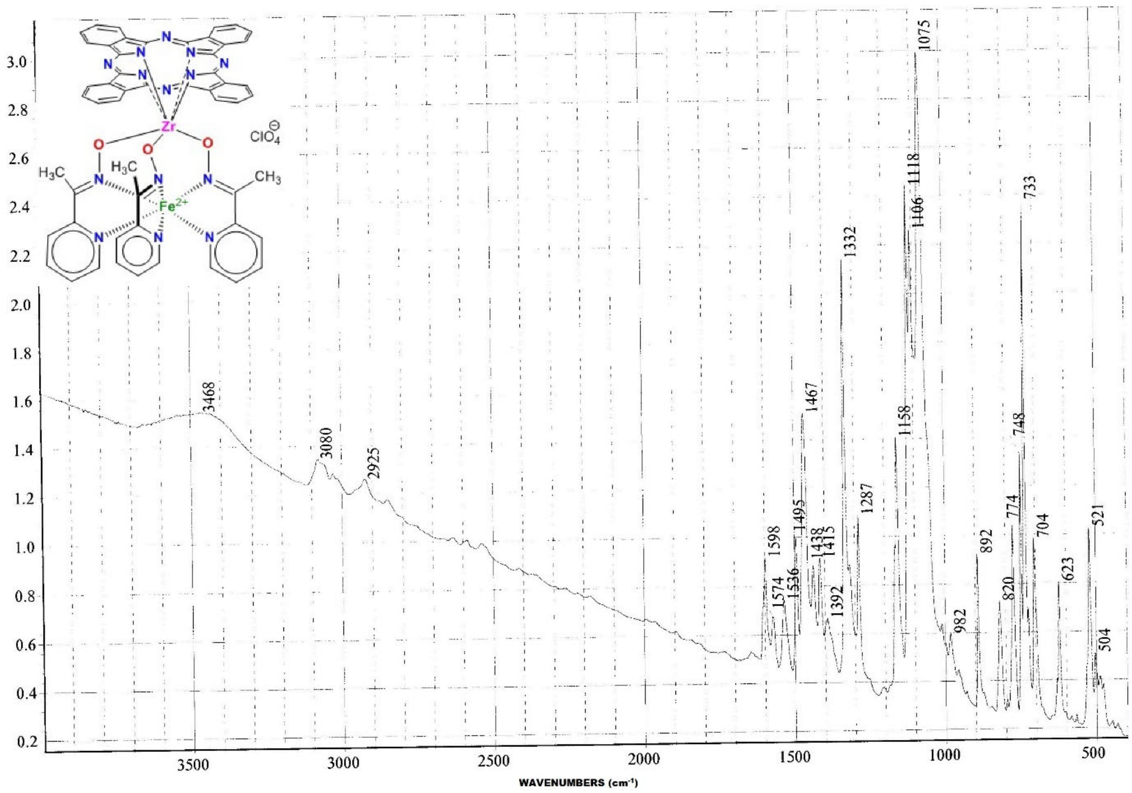

Figure S3. IR spectrum of **11**.

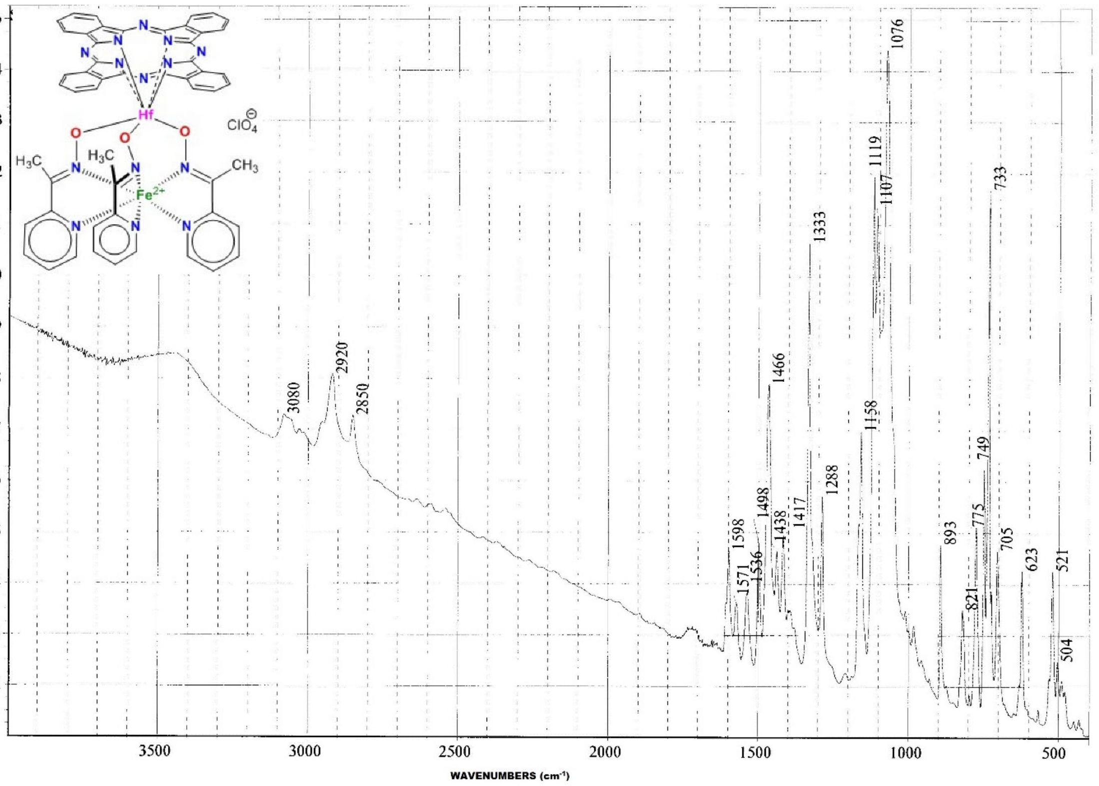

Figure S4. IR spectrum of **12**.

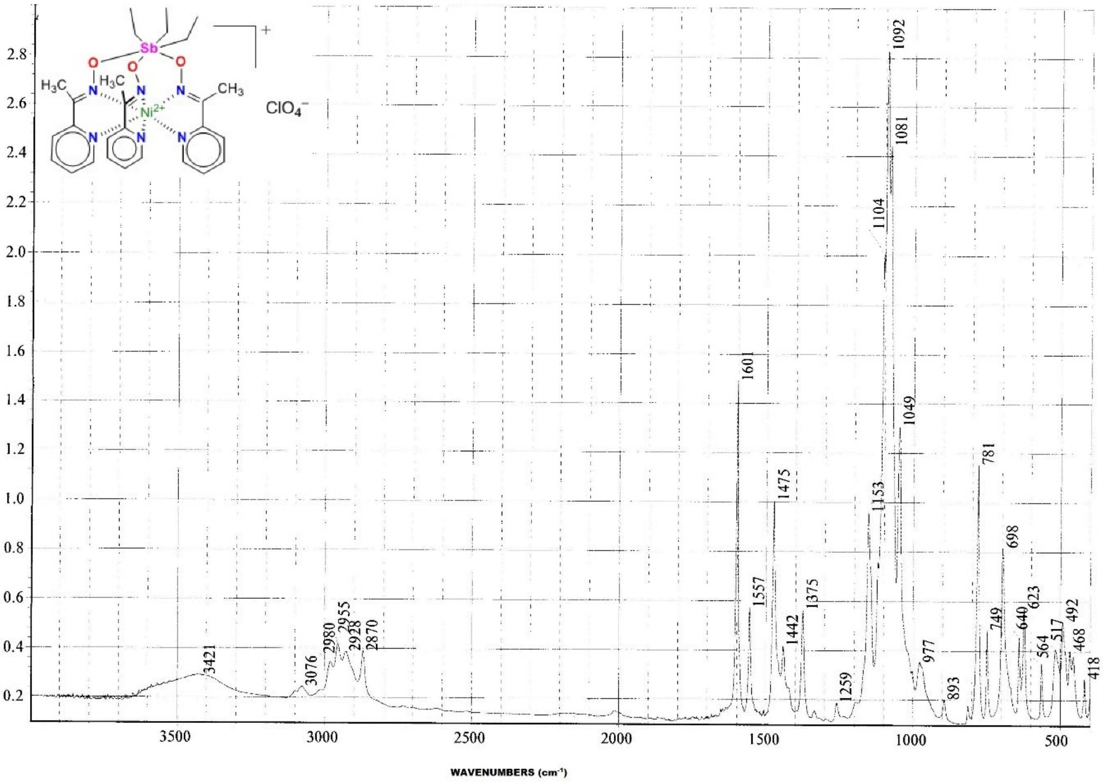

Figure S5. IR spectrum of **13**.

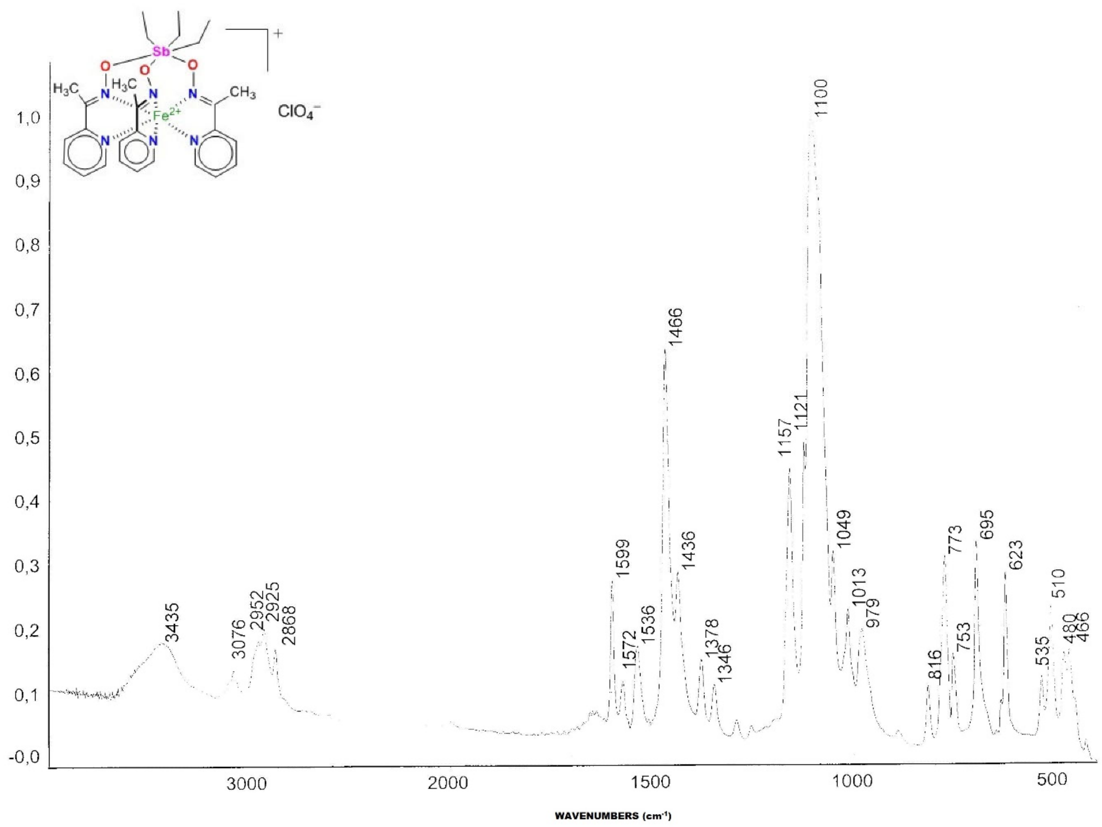

Figure S6. IR spectrum of **14**.

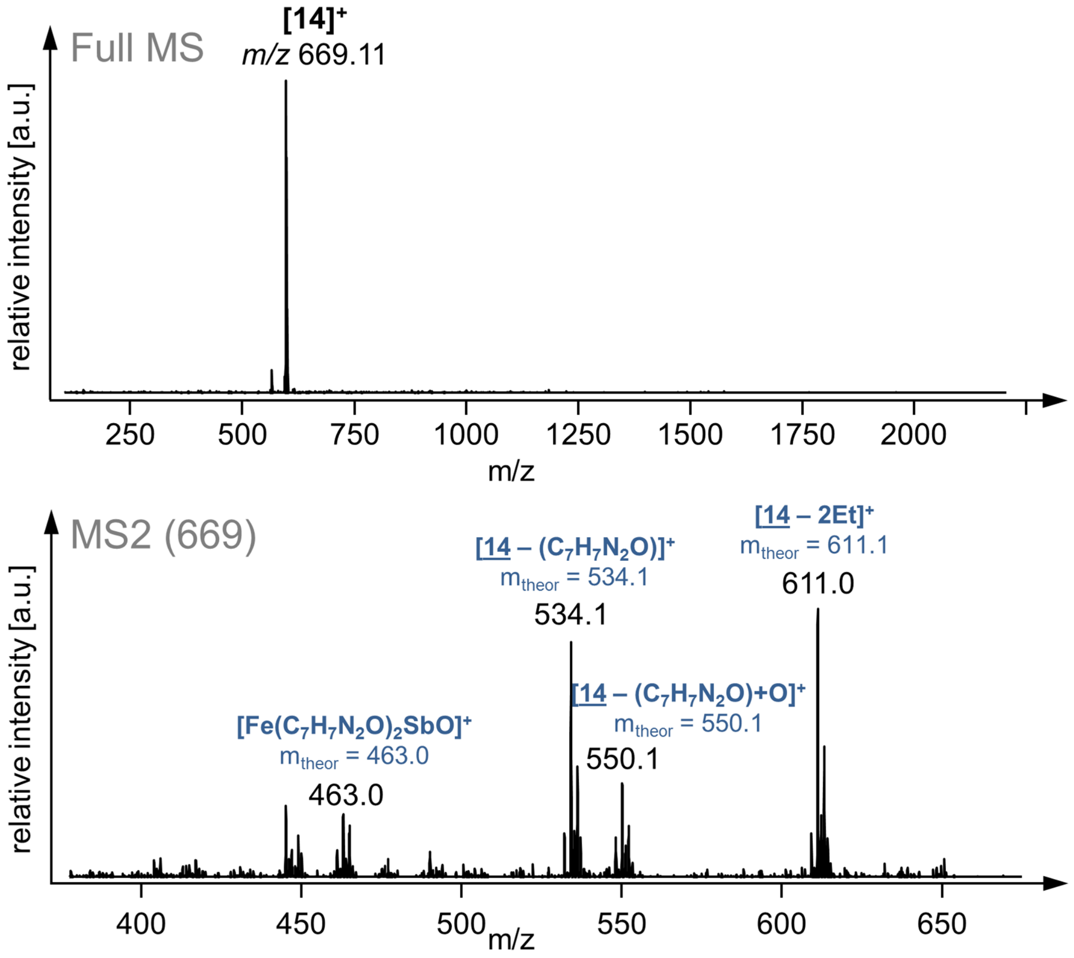

Figure S7. The full mass spectra (top) and the associated fragmentation experiment MS2 (bottom) upon isolation of the given precursor are shown for the synthetic precursor **14** (Fe-Sb). The corresponding Ni-Sb complex **13** did not yield interpretable fragmentation spectra.

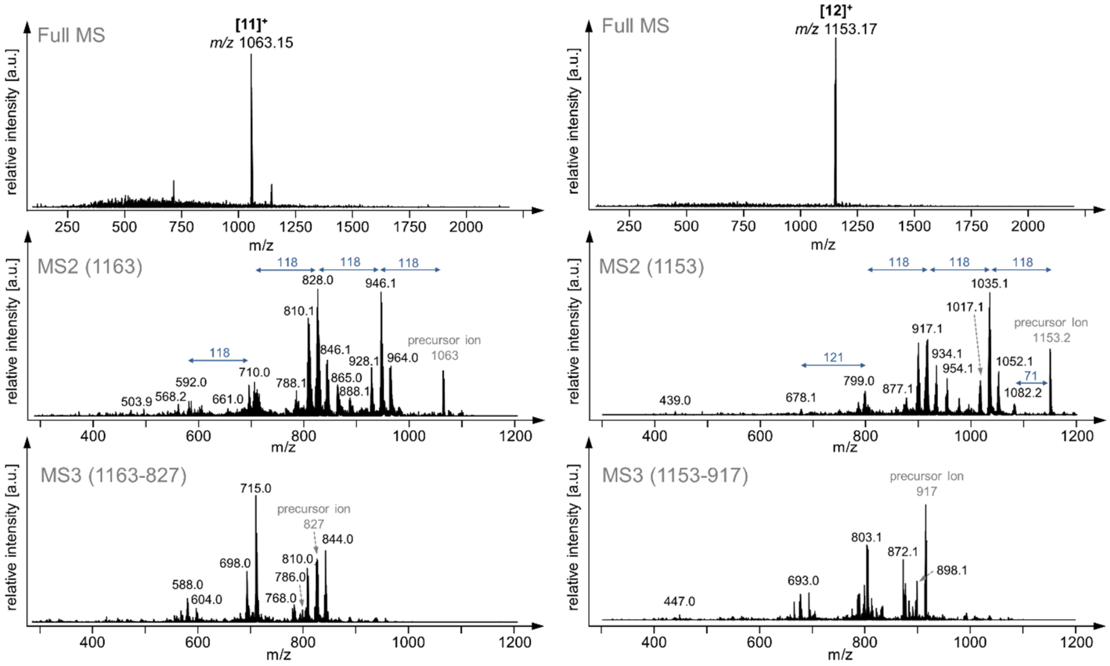

Figure S8. The full mass spectra (top) and their associated fragmentation experiments MS2 (middle) and MS3 (bottom) upon isolation of the given precursor are shown for **11** (Zr-Fe) and **12** (Hf-Fe).

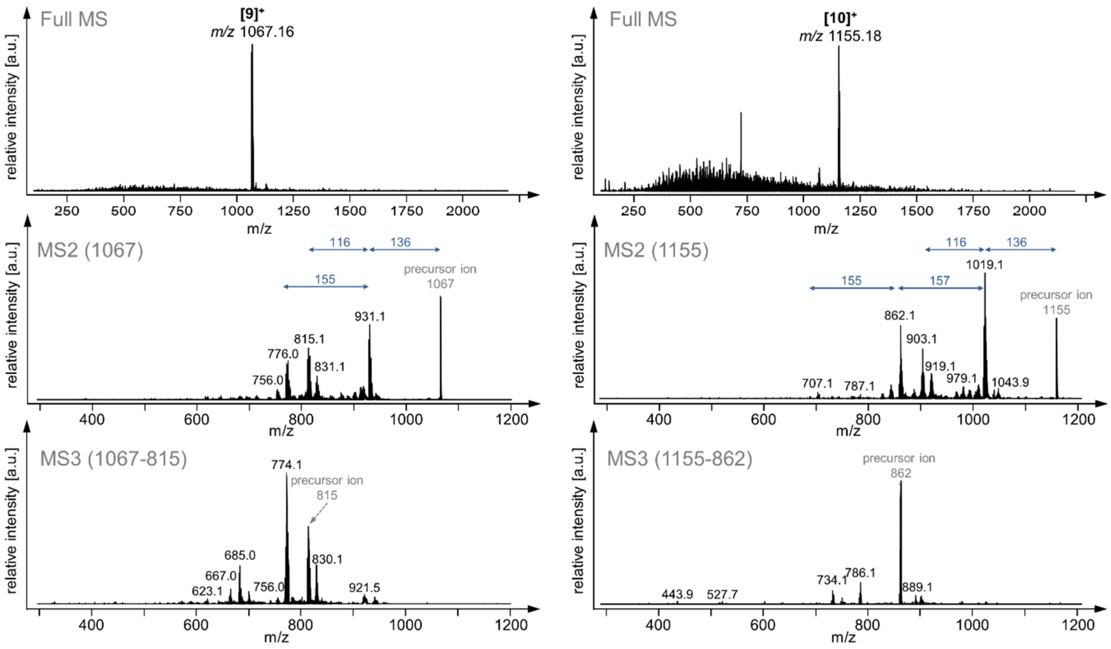

Figure S9. The full mass spectra (top) and their associated fragmentation experiments MS2 (middle) and MS3 (bottom) upon isolation of the given precursor are shown for **9** (Zr-Ni) and **10** (Hf-Ni).

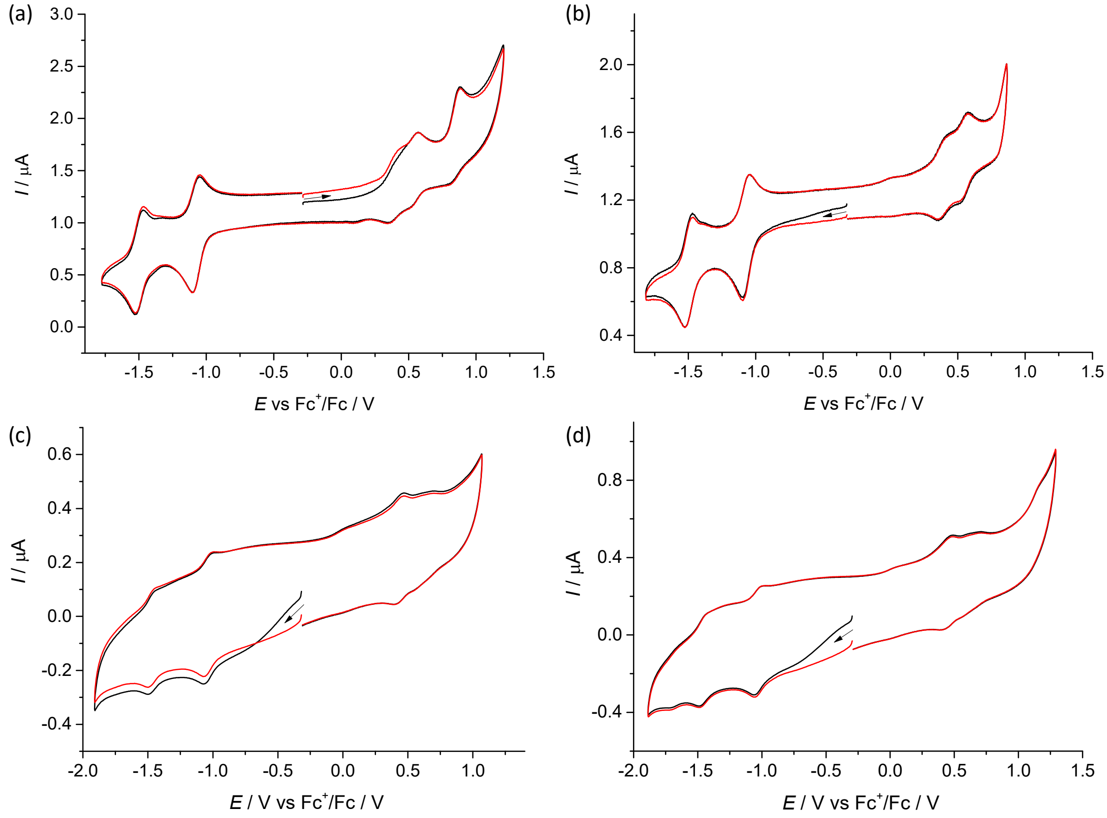

Figure S10. Cyclic voltammograms for complexes (a) **9**, (b) **10**, (c) **11** and (d) **12**, in ACN/*n*-Bu_4NPF_6 (Pt-disc working electrode, scan rate 100 mV s^{-1}).

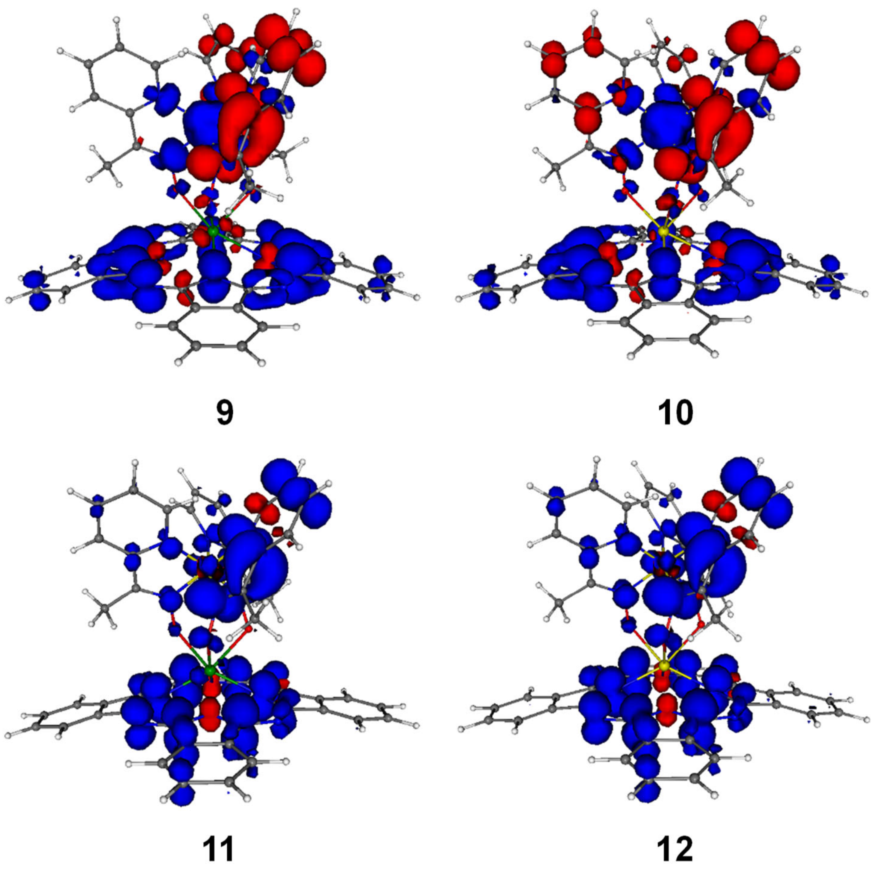

Figure S11. B3LYP/LanL2DZ Spin density distributions in double-reduced forms of complexes **9–12**. The isovalue is set to ±0.002. Energy differences between the singlet and triplet electronic states ($\Delta E_{S\text{–}T}$) are 0.035, 0.034, 0.0035 and 0.0034 a.u. for complexes **9**, **10**, **11** and **12**, respectively.

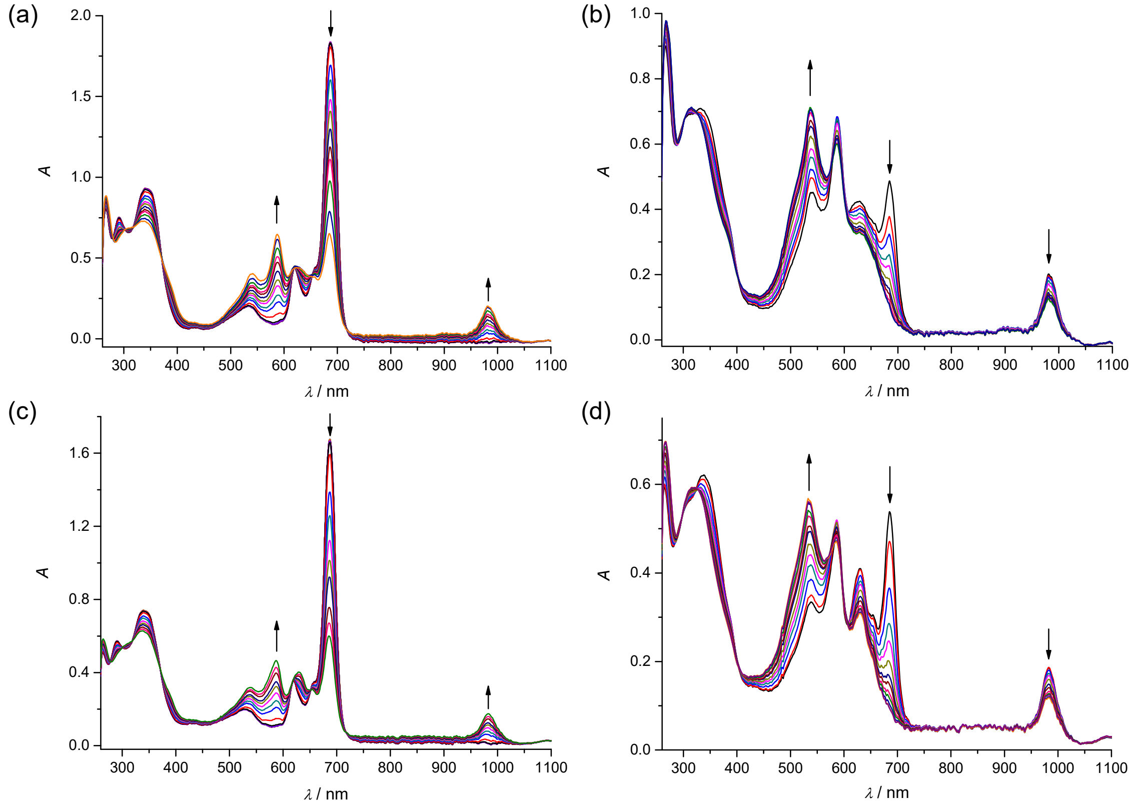

Figure S12. In situ UV-vis-NIR spectroelectrochemistry of complex **11 (Fe-Zr)** in the region of (a) the first and (b) the second cathodic peak, as well as of complex **12 (Fe-Hf)** in the region of (c) the first and (d) the second cathodic peak, all in ACN/*n*-Bu_4NPF_6 in a thin layer honeycomb spectroelectrochemical cell.

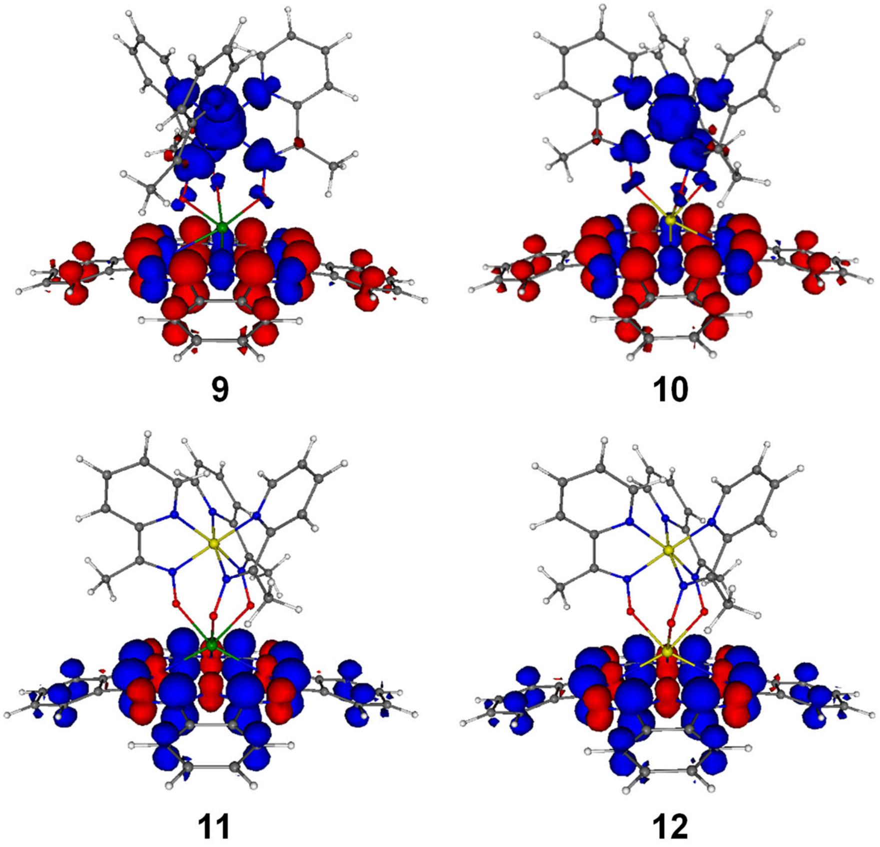

Figure S13. B3LYP/LanL2DZ spin density distributions in 1e-oxidized forms of complexes **9–12**. The isovalue is set to ±0.002.

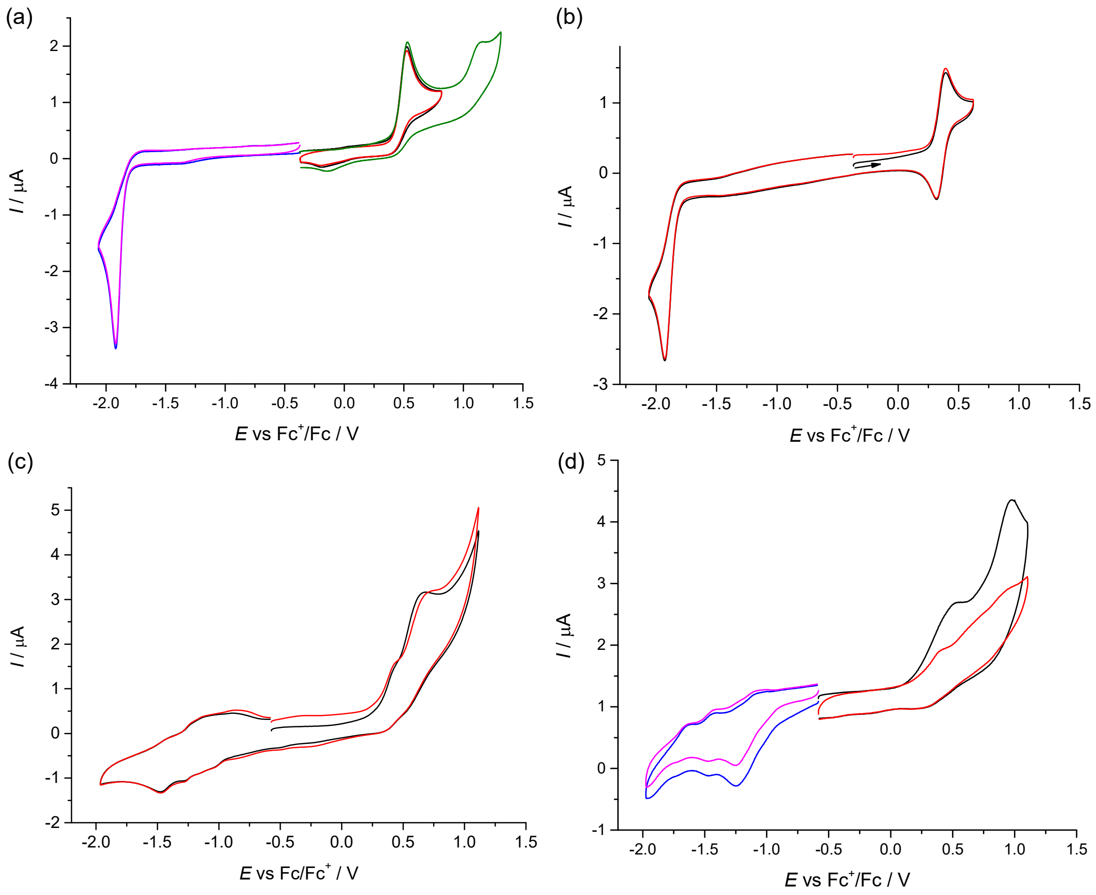

Figure S14. Cyclic voltammograms for the corresponding molecular precursors **13–16** of investigated hybrid Zr/Hf-phthalocyaninate-capped metal(II) tris-pyridineoximates **9–12** in ACN/*n*-Bu_4NPF_6 solutions (Pt-disc working electrode, scan rate 100 mV s^{-1}): (a) **13**, (b) **14**, (c) **15** and (d) **16**.

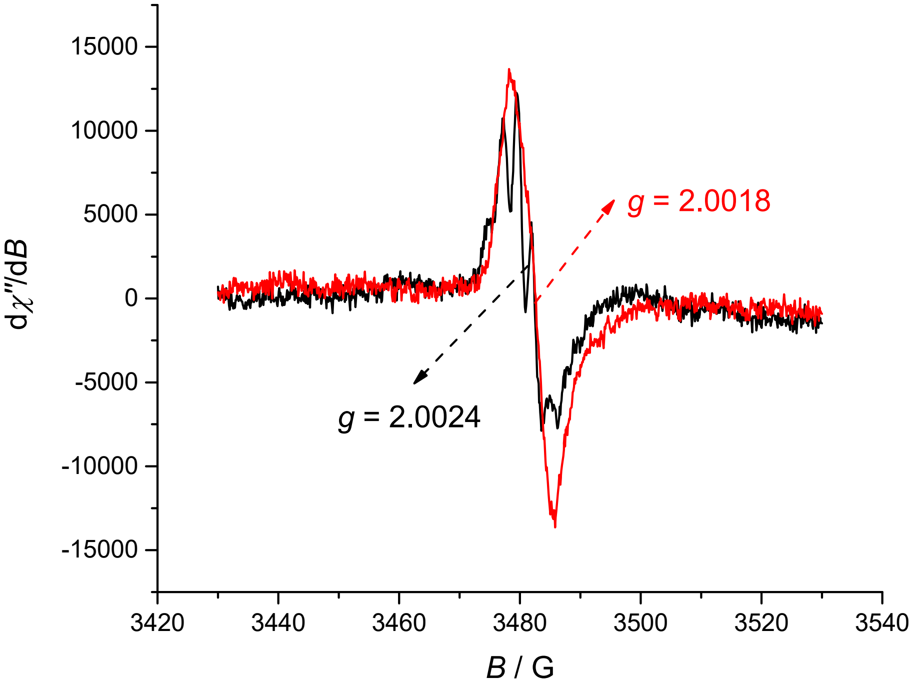

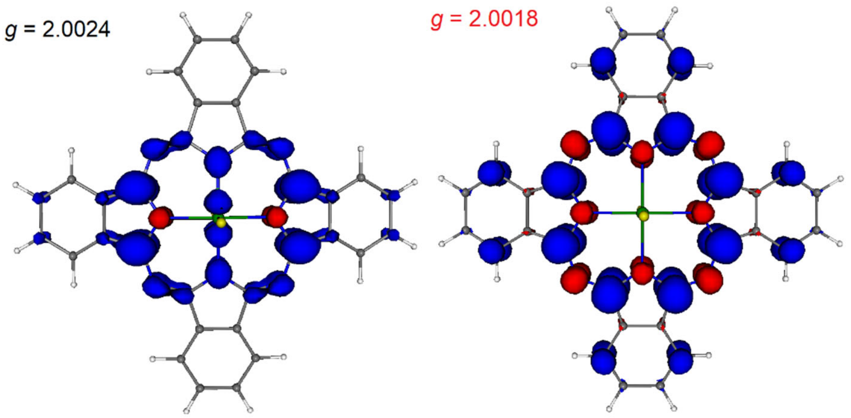

Figure S15. Top: EPR spectrum of 1e-reduced precursor **15** (black trace) and 1e-oxidized **15** (red trace) in ACN/*n*-Bu_4NPF_6. Bottom: B3LYP/LanL2DZ spin density distributions in 1e-reduced (left) and 1e-oxidized (right) precursor **15**. The isovalue is set to ±0.002.

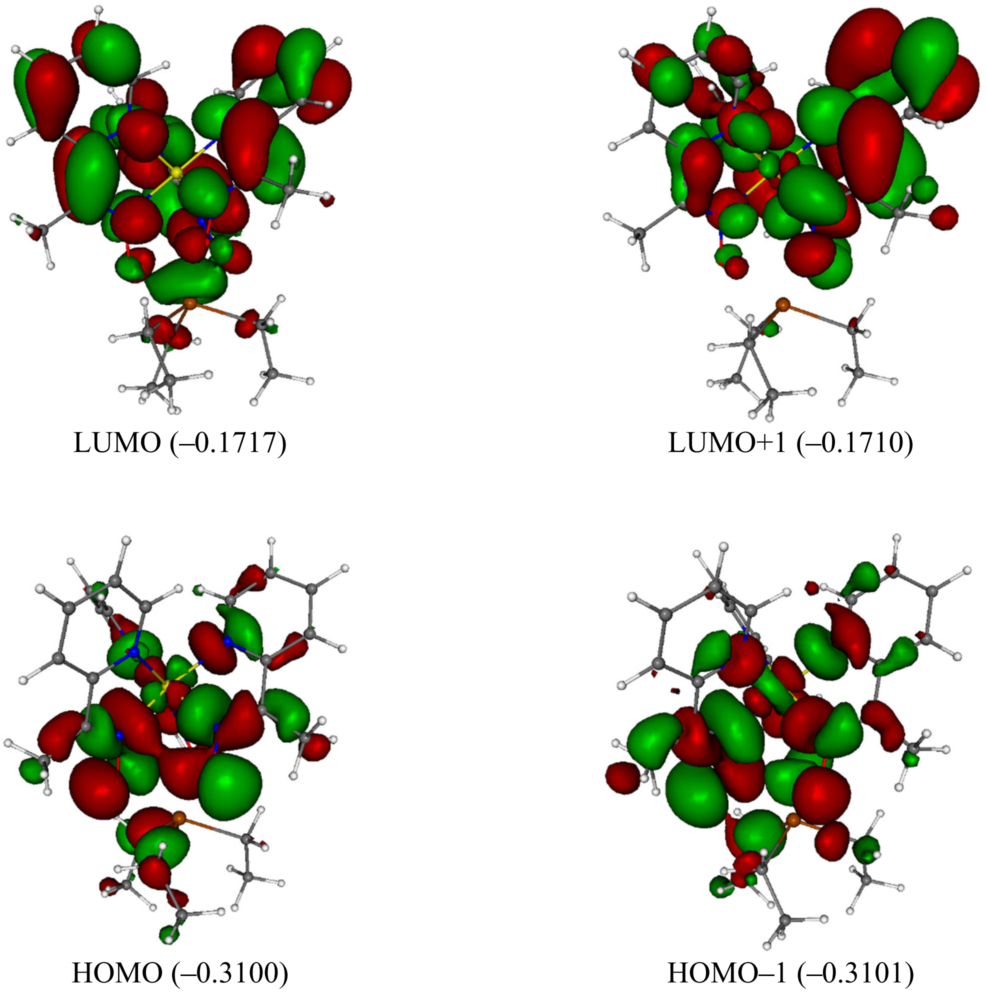

Figure S16. B3LYP/LanL2DZ HOMO–1, HOMO, LUMO, and LUMO+1 orbitals of the precursor **13**, the corresponding relative orbital energies in a.u. are given in parenthesis. The isovalue is set to ±0.02.

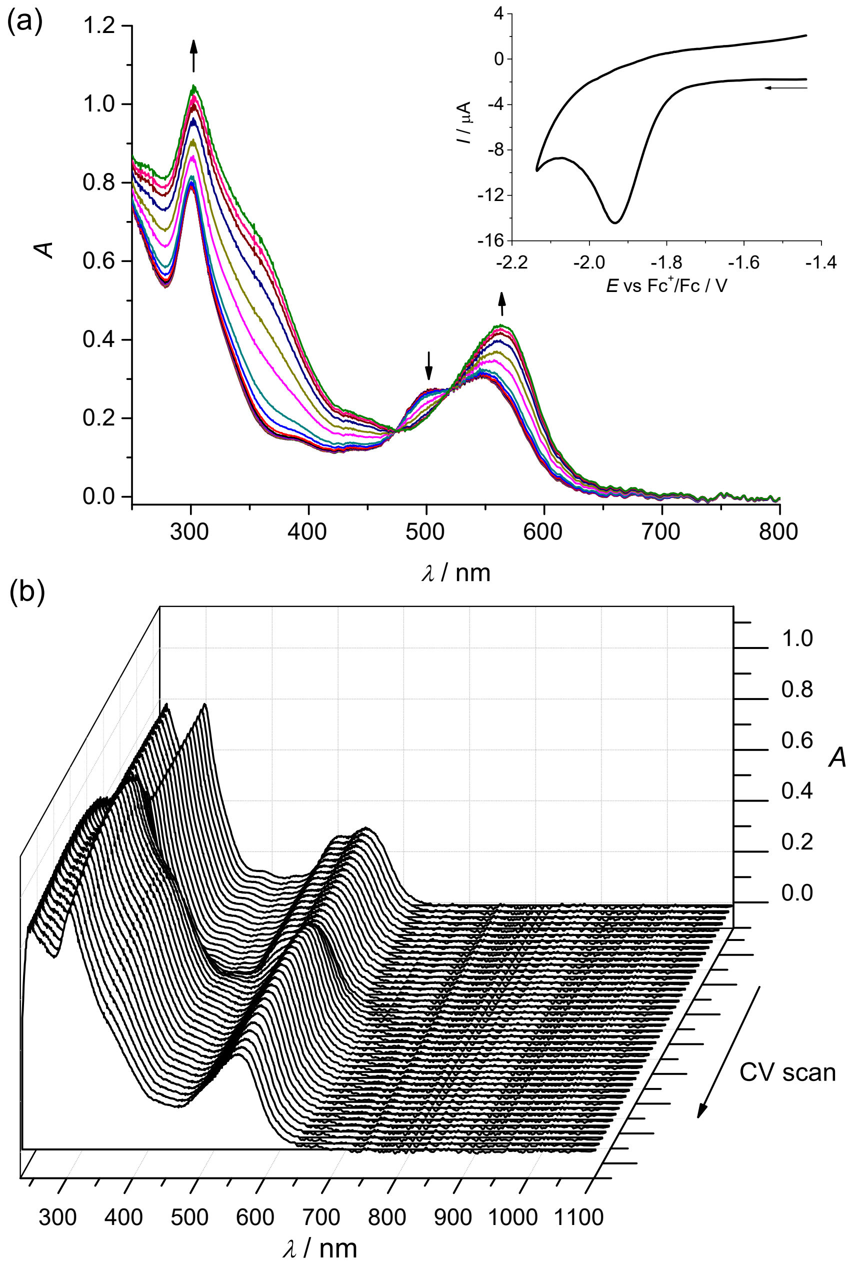

Figure S17. Evolution of UV-vis-NIR spectra in (a) 2D (forward scan) and (b) 3D projection during cyclic voltammetry (see the corresponding voltammogram in Inset of (a), scan rate 10 mV s^{-1}) of **14** in the region of the first reduction peak in ACN/*n*-Bu_4NPF_6.
